# Complete Genome Sequence of *Paenibacillus polymyxa* SQR-21, a Plant Growth-Promoting Rhizobacterium with Antifungal Activity and Rhizosphere Colonization Ability

**DOI:** 10.1128/genomeA.00281-14

**Published:** 2014-04-10

**Authors:** Shuqing Li, Dongqing Yang, Meihua Qiu, Jiahui Shao, Rong Guo, Biao Shen, Xihou Yin, Ruifu Zhang, Nan Zhang, Qirong Shen

**Affiliations:** a National Engineering Research Center for Organic-Based Fertilizers, Nanjing Agricultural University, Nanjing, China; b Jiangsu Collaborative Innovation Center for Solid Organic Waste Utilization, Nanjing Agricultural University, Nanjing, China; c Key Laboratory of Plant Nutrition and Fertilization in Low-Middle Reaches of the Yangtze River, Ministry of Agriculture, Nanjing, Nanjing Agricultural University, Nanjing, China; d Department of Pharmaceutical Sciences, Oregon State University, Corvallis, Oregon, USA

## Abstract

Here we report the complete genome sequence of a plant growth-promoting rhizobacterium (PGPR), *Paenibacillus polymyxa* SQR-21, which consists of one circular chromosome of 5,828,438 bp with 5,024 coding sequences (CDS). The data presented highlight multiple sets of functional genes associated with its plant-beneficial characteristics.

## GENOME ANNOUNCEMENT

*Paenibacillus polymyxa* (formerly *Bacillus polymyxa*), a Gram-positive, sporulating bacterium, is considered to be a plant growth-promoting rhizobacterium (PGPR) (1, 2). Originally isolated from various environmental samples (3, 4), *P. polymyxa* has been widely applied in agriculture, industry, medicine, and environmental remediation (5–12). Thus far, only four *P. polymyxa* genomes, those of *P. polymyxa* E681 (NC_014483) (13), *P. polymyxa* SC2 (NC_014622) (14), *P. polymyxa* M1 (NC_017542) (1), and *P. polymyxa* CR1 (NC_023037) (15), have been completely sequenced. *P. polymyxa* SQR-21 was naturally isolated from watermelon rhizosphere. As an outstanding PGPR strain with the ability to produce various antibiotics (16, 17) and colonize rhizospheres (18), SQR-21 has been widely exploited in commercial biofertilizers for plant growth promotion and biological control of soilborne plant pathogens (19, 20). Here, we report the whole-genome sequence of SQR-21 in order to better elucidate its plant-beneficial characteristics and promote its agricultural applications.

Whole-genome sequencing of *P. polymyxa* SQR-21 was performed with Roche 454 sequencing technology. The constructed library was sequenced by the GS FLX Titanium series chemistry. A total of 1,000,000 sequence reads with average read lengths of 350 to 450 bp (resulting in up to 400 Mb of sequence data) were obtained, representing an average of 70-fold coverage of the genome. The reads were assembled into 19 scaffolds, and the 54 sequence gaps both within and between the scaffolds were filled by sequencing PCR products using an ABI 3730 capillary sequencer.

The complete genome of *P. polymyxa* SQR-21 is composed of a 5,828,438-bp circular chromosome, with a mean GC content of 45.64%. Based on the genomic data, 5,024 coding sequences (CDS) were predicted by GeneMark (21) and annotated by a BLAST tools search against the GenBank nonredundant protein database (NR), Kyoto Encyclopedia of Genes and Genomes (KEGG), and Clusters of Orthologous Groups (COG). In addition, 111 tRNA loci and 13 rRNA operons were identified with the tRNAscan-SE (22) and RNAmmer 1.2 (23) servers, respectively.

*P. polymyxa* SQR-21 harbors four nonribosomal peptide synthetase (NRPS) gene clusters, including *pmx* and *fus* gene clusters responsible for biosynthesis of polymyxin and fusaricidin, respectively, which reveal high similarities to the published gene clusters (24–26). One polyketide synthetase (PKS) gene cluster, three hybrid PKS-NRPS gene clusters, and three gene clusters relevant to lantibiotic biosynthesis are also present in the SQR-21 genome. Several genes involved in plant growth promotion, including genes responsible for production of indole-3-acetic acid (IAA), 3-hydroxy-2-butanone (acetoin), and 2,3-butanediol, as well as phytase, were identified in the SQR-21 genome. In addition, the genome harbors a set of genes encoding extracellular enzymes involved in the degradation of plant-derived polysaccharides, including xylanase, glucanase, and chitinase. Given the strain specificity and application in agricultural production, the complete genome sequence of *P. polymyxa* SQR-21 provides useful information for both basic and applied research, which also facilitates the understanding of the functions and evolutions of the *Paenibacillus polymyxa* genome.

### Nucleotide sequence accession number.

The complete sequence of *Paenibacillus polymyxa* SQR-21 has been deposited in NCBI’s GenBank under the accession number CP006872.
